# Intra- and Inter-Frequency Brain Network Structure in Health and Schizophrenia

**DOI:** 10.1371/journal.pone.0072351

**Published:** 2013-08-26

**Authors:** Felix Siebenhühner, Shennan A. Weiss, Richard Coppola, Daniel R. Weinberger, Danielle S. Bassett

**Affiliations:** 1 Department of Physics, University of California Santa Barbara, Santa Barbara, California, United States of America; 2 Neuroscience Center, University of Helsinki, Helsinki, Finland; 3 Department of Neurology, Columbia University, New York, New York, United States of America; 4 MEG Core Facility, National Institute of Mental Health, Bethesda, Maryland, United States of America; 5 Genes, Cognition and Psychosis Program, Clinical Brain Disorders Branch, National Institute of Mental Health, Bethesda, Maryland, United States of America; 6 Lieber Institute for Brain Development, Johns Hopkins Medical Campus, Baltimore, Maryland, United States of America; 7 Sage Center for the Study of the Mind, University of California Santa Barbara, Santa Barbara, California, United States of America; Wake Forest School of Medicine, United States of America

## Abstract

Empirical studies over the past two decades have provided support for the hypothesis that schizophrenia is characterized by altered connectivity patterns in functional brain networks. These alterations have been proposed as genetically mediated diagnostic biomarkers and are thought to underlie altered cognitive functions such as working memory. However, the nature of this dysconnectivity remains far from understood. In this study, we perform an extensive analysis of functional connectivity patterns extracted from MEG data in 14 subjects with schizophrenia and 14 healthy controls during a 2-back working memory task. We investigate uni-, bi- and multivariate properties of sensor time series by computing wavelet entropy of and correlation between time series, and by constructing binary networks of functional connectivity both within and between classical frequency bands (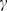, 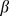, 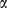, and 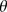). Networks are based on the mutual information between wavelet time series, and estimated for each trial window separately, enabling us to consider both network topology and network dynamics. We observed significant decreases in time series entropy and significant increases in functional connectivity in the schizophrenia group in comparison to the healthy controls and identified an inverse relationship between these measures across both subjects and sensors that varied over frequency bands and was more pronounced in controls than in patients. The topological organization of connectivity was altered in schizophrenia specifically in high frequency 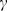 and 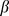 band networks as well as in the 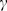-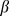 cross-frequency networks. Network topology varied over trials to a greater extent in patients than in controls, suggesting disease-associated alterations in dynamic network properties of brain function. Our results identify signatures of aberrant neurophysiological behavior in schizophrenia across uni-, bi- and multivariate scales and lay the groundwork for further clinical studies that might lead to the discovery of new intermediate phenotypes.

## Introduction

Synchronous oscillatory neuronal activity is thought to form a neurophysiological network over which the brain processes information during cognitive processes [1], [2]. Coherent neuronal communication relies on neurotransmission dynamics dictated by major neurotransmitters like dopamine, GABA, glutamate and acetylcholine systems, known to be altered in schizophrenia [3]–[5].

Indeed, a hallmark of schizophrenia is the complex pattern of abnormal increases and decreases in resting state and task-based connectivity evident between large-scale brain regions [6]–[8]. To quantify this *dysconnectivity* [4], [9] at the level of the whole brain, network theoretical tools originally developed in the social sciences have been applied to neuroimaging data. Results demonstrate complex dysconnectivity profiles of the brain's anatomy [10]–[12] and function [5], [13]–[20] in people with schizophrenia [21].

Such dysconnectivity profiles, evidenced by altered brain activity and connectivity in schizophrenia, are accompanied by altered cognitive behavior. Due to the heterogeneity of the disease, some symptoms including both positive (hallucinations, delusions and thought disorder) and negative (e.g. impaired emotional response and social interaction, avolition, anhedonia) symptoms, vary strongly among patients. However, cognitive impairments in executive functions (such as the manipulation of transiently stored information [22]) and working memory (e.g., the ability to transiently maintain and manipulate a limited amount of information in order to guide thought or behavior) are typically altered to varying degrees in virtually all patients. These cognitive impairments appear early and change little over time, and are therefore thought to lie at the core of the disease, contributing to the development of clinical symptoms [23], [24]. Both altered memory and executive planning have been linked to the neurochemistry of dopamine, GABA, glutamate and acetylcholine systems and subsequently to aberrant (NMDAR)–mediated synaptic plasticity [3]–[5].

Correlations between brain network organization and cognitive or behavioral variables in schizophrenia are expected [25] but quantitative evidence for such a relationship is still rare. Network-based analyses of task-based *functional connectivity* could potentially provide unique insight into the neurophysiological underpinnings of altered cognitive function. Indeed, such approaches have been used to link brain network organization in healthy cohorts to a range of cognitive functions including attention [26], [27], memory [14], [28]–[30], learning [31]–[34], and emotion [35] and language processing [36]. Recent studies in schizophrenia cohorts have further demonstrated that the organization of task-based functional brain networks can be linked both to the efficacy of auditory rehabilitation efforts [16] and to working memory performance [5], [14], [30], [37]. These findings suggest that the characterization of the dynamic network of task-based synchronous brain activity might be a powerful tool to study cognitive impairments in schizophrenia, providing insight into the neurobiological mechanisms of the disease. However, many of these studies have several potential limitations, such as using only a small set of network diagnostics and constructing a single functional network from a large array of data.

In this study, we provide an expanded examination of the functional brain network architecture in people with schizophrenia estimated from magnetoencephalography (MEG) data acquired during the performance of a working memory N-back task. We apply a multiresolution approach [20] which builds on previous work in the identification of neurophysiological markers of schizophrenia in resting state fMRI data [19], [20], [38]. These studies together hypothesize that neurophysiological alterations in schizophrenia will manifest themselves as a complex hierarchy of signatures across univariate, bivariate, and multivariate statistical measurements. By examining these measurements in concert, we are able identify scale-specific and scale-generic properties of abnormalities which could have implications for understanding putative neurobiological mechanisms.

In this multiscale approach, we focus on entropy (univariate), functional connectivity (bivariate), and network topology (multivariate). More specifically, we examine gross measurements of functional connectivity and explore their link to single time series variability, a relationship previously identified in resting state fMRI data [19], [20], [38]. To quantitatively characterize functional brain network structure, we examine 12 binary network diagnostics across a wide range of densities and employ a recently developed statistical technique (functional data analysis) to identify significant group differences. To detect time-varying network structure and to increase statistical robustness, we construct an ensemble of networks for each individual using data extracted from 66 separate trials.

Finally, in a novel extension of the analysis of functional brain network structure in the classical frequency bands of brain activity, we examine functional networks constructed from the interactions between frequency bands. Inter-frequency networks are particularly interesting in the context of our examination of the N-back task because cross-frequency interactions are thought to facilitate memory function [39] by enabling the cross-function integration of information over spectrally distinct processing streams [40]. Furthermore, evidence suggests that cross-frequency interactions are altered in schizophrenia, and the strength of these alterations has been linked to genetic risk factors for the disease [41]. Based on a prior study showing regional specificity of increased and decreased cross-frequency coupling in schizophrenia [41], we expected that patterns of cross-frequency connectivity would be significantly altered in the disease, supporting the hypothesis that overall processing and integration of information is impaired in schizophrenia.

Using these tools, we uncover an extensive pattern of altered network structure and network dynamics in people with schizophrenia, laying the groundwork for further studies that could potentially lead to the identification of intermediate phenotypes [42] and development of diagnostic biomarkers for the disease [21], [43].

## Materials and Methods

### Participants

14 healthy volunteers and 14 people diagnosed with schizophrenia spectrum disorders (according to the Diagnostic and Statistical Manual of Mental Disorders IV criteria) took part in the Clinical Brain Disorders Branch/National Institute of Mental Health Genetic Study of Schizophrenia (National Institutes of Health Study Grant NCT 00001486, Daniel R. Weinberger, principal investigator). None of the healthy volunteers had qualitative structural magnetic resonance imaging abnormalities or history of psychiatric illness, depression, or loss of consciousness. All patients were chronic (mean duration of illness 12.7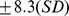) outpatients, of which 12 were receiving antipsychotic medication at the time of the study (see Table S2 in the supplementary information (SI)); none of the healthy volunteers were taking psychoactive medication. Mean(STD) PANSS scores for the patients were 15.5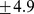 on the positive symptoms scale, 18.3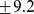 on the negative symptoms scale and 34.4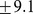 on the general psychopathology scale. For a full tabulation of PANSS scores for participants, see Table S1 in the SI. Subjects were matched for sex (9 males, 5 females in each group) and did not differ significantly in age. The mean age of the healthy control group was 30 years 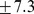 (SD), and that of the patient group was 33 years 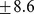 (SD).

### Ethics statement

The protocol for this experiment was approved by the National Institute of Mental Health Institutional Review Board. All healthy participants gave informed consent in writing. We assessed the capacity of patients to provide consent for participation in studies on a case-by-case basis. The Human Subjects Protection Unit (HSPU) at the National Institutes of Health performed the capacity assessment and provided consent monitoring as needed. For subjects without the capacity to provide informed consent, consent was instead obtained from the holder of the Durable Power of Attorney (DPA). For any patient who did not have legal status (regardless of capacity) to provide informed consent, the guardian authorized the research by signing the consent form. When such surrogate consent was obtained, the patient indicated assent by also signing the consent form.

### Data acquisition

MEG data were acquired at the National Institute of Mental Health in Bethesda, MD. The experimental paradigm was a visual 2-back working-memory task using numerical stimuli ranging from 1 to 4 as previously described [44]. Earlier studies using the same task demonstrated abnormal behavioral and neuroimaging associations with schizophrenia [45]–[47]. Visual stimuli (numbers in the range from 1 to 4) were presented visually, each for 500 msec. After the presentation of the third stimulus, the subject was asked to respond within a 1300 ms stimulus-free period by pressing a button to indicate the identity of the stimulus seen 2 stimuli previously. After the presentation of the fourth stimulus (which also comprised 500 ms), the subject was asked to respond within the same 1300 ms stimulus-free period by pressing a button to indicate the identity of the stimulus seen 2 stimuli previously. This combination of presentation (500 ms) and response (1300 ms) periods continued for the next 7 stimuli, for a total of 11 stimulus-response periods, each 1800 ms in duration. Each subject performed 6 blocks, each composed of 11 stimulus-response periods. During the performance of this task, data were recorded using a 275-channel CTF system (VSM MedTech) with a sampling rate of 600 Hz.

### Data preprocessing

We used MATLAB [48] and FieldTrip software [49], for preprocessing of the data. Eye blinks were identified and removed from the raw data by an automated process using a threshold detection function, followed by a signal space separation based on principal components analysis (PCA). Raw data were mean corrected and filtered to attenuate background low-frequency noise and line noise at 60 Hz by using a 0.3-Hz-width filter.

We obtained an estimate of head movement during the recording by calculating the difference (in centimeters) between the headcoil position prior to and following the recording. We used a group-level cutoff criteria of an average of 0.5 cm or smaller deviation in position pre-post. Both groups met the criteria. Additional corrections for head movement were not employed for several reasons. As highlighted in [50], head movement artifact correction methods [51]–[54] are inherently unstable to large movements and may also fundamentally alter the MEG time series adversely, possibly due at least in part to the assumptions of time series stationarity. Because we do not perform source reconstruction, we follow the advice outlined in [50] by examining the raw data for single trials in sensor space without head movement correction. This is considered a statistically conservative approach, prone to Type II (false negative) rather than Type I (false positive) errors [50].

The axial gradiometers of the CTF machine have source profiles that include information from a wide spatial range, strictly limiting the interpretation of the anatomical location of results. To retain the inherent correlation structure of a network of interacting brain regions while gaining localization specificity, we transformed the data into planar space. Using Fieldtrip, the planar transform was applied to the data by using the function *ft_megplanar*. This function represents a transformation from the radial gradient measured by the CTF system to tangential gradient configuration, aimed at improving localization specificity [49].

For trial-by-trial analysis, the time series were cut into epochs of 1800 ms, composed of the presentation of the third stimulus in a set (500 ms in duration) and the stimulus-free response period (duration 1300 ms). We refer to these 1800 ms epoch as *trials* throughout the remainder of the manuscript. Because each subject performed 6 blocks of 11 sets of stimuli, we investigated a total of 66 trials per subject.

### Wavelet decomposition

We focused our investigation on frequency-band-specific oscillations in the MEG signal by employing a wavelet analysis. Time series were resampled to 120 Hz to constrain the frequency bands of the wavelet transform to roughly conform to the classical frequency bands [14]. Each frequency band of interest was isolated by applying the maximal overlap discrete wavelet transform to each time series [55]. In line with previous work, we used the Daubechies 4 wavelet [14]. Wavelet scale 1 (30–60 Hz) roughly corresponds to the 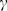 band, scale 2 (15–30 Hz) to 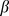, scale 3 (8–15 Hz) to 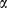, and scale 4 (4–8 Hz) to 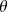. Due to the relatively short trial length (1.8 s), we did not examine frequencies below 4 Hz. Due to the sampling frequency (120 Hz), we had more power to estimate coefficients in the lower frequency range of wavelet scale 1 than the upper frequency range and we therefore refer to this scale throughout the remainder of the manuscript as low 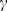 or 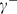.

### Functional connectivity and complexity estimation

Before constructing functional brain networks, we characterized each sensor's wavelet time series and the pairwise relationship between sensor time series. To quantify the complexity of the time series, we computed the Shannon wavelet entropy [20], [56], [57], which we describe in detail in the SI. To investigate functional connectivity between sensors, we calculated the mutual information (MI), which is particularly appropriate for estimating interactions that encompass a narrow frequency band (e.g., a wavelet scale) [58]. Each pairwise MI calculation utilized 216 time points (1800 ms sampled at 120 Hz), an epoch length similar to that used in previous neuroimaging studies [59]–[61]. Due to the finite length of data, biases and random errors can not be ruled out [62], but these effects should be strongly mitigated by our utilization of multiple trials to identify network signatures of the cognitive processes common to all epochs.

We represent pairwise sensor MI both within and between frequency bands as sensor-by-sensor (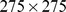) functional connectivity matrices. To construct intra-frequency functional connectivity matrices, we calculated the MI between the time series in frequency band 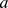 of all possible pairs of sensors to create the weight matrix 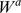. To construct inter-frequency functional connectivity matrices, the process was similar: for each pair of frequency bands 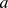 and 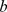, we calculated the MI between the time series of sensor 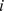 in frequency 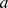 and sensor 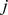 in frequency 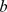 for all possible pairs of sensors 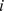 and 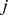. This resulted in the weight matrix 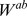. The pairwise mutual information values in both the intra- and inter-frequency matrices were normalized according to Strehl and Ghosh [63], ensuring that the values of the elements of 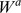 and 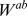 were in the range 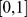. Through this process, we created 4 intra-frequency functional connectivity matrices (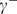, 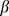, 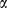, and 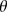) and 6 inter-frequency functional connectivity matrices (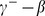, 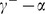, 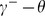, 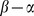, 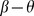, 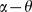) per subject for each of the 66 trials (660 total networks per subject).

The magnitude of functional connectivity is quantified using the *network strength* (hereafter referred to simply as *strength*) of sensor 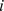, which is defined mathematically as the average MI between sensor 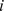 and all other sensors in the network [15], [20], [64]–[69]: 
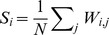
. The strength of the network is defined as the mean strength of all sensors: 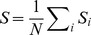.

### Network properties

To quantitatively characterize the topology of the intra- and inter-frequency functional connectivity matrices, we construct sets of binary networks summarizing the interactions (edges) between sensors (nodes). A *binary network* can be represented mathematically by an adjacency matrix 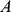, whose entries 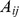 are either 0 or 1, indicating the absence or presence of an edge respectively. Binary networks can be obtained by thresholding a given functional connectivity matrix in several ways [20], [70]. Here we employ the technique of cumulative thresholding, a procedure in which a threshold is applied to the weighted functional connectivity matrix 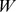 to retain a given percent of strongest connections. Weights (e.g., MI values) that pass this threshold were set to a value of 1 in the adjacency matrix 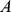, while those that did not pass this threshold were set to a value of 0 in the adjacency matrix 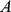.

The percent of nonzero elements of 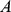 is also known as the network density, or network cost 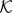. We can employ a range of thresholds to probe the topological organization of a network over a range of network densities. Using a high threshold, we obtain a sparse matrix representing the topology of the strongest functional associations between sensors, while using a lower threshold, we obtain a denser matrix representing the topology of functional associations over a wider range of strengths. In this study, we employed a set of thresholds enabling us to examine corresponding sets of networks with densities ranging from 0 (no connections present) to 0.5 (half of the possible number of connections present) in steps of 0.01. We refer to this network density range 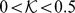 as the cost regime of interest. Our choice to examine network organization over a large range of network density values is supported by previous studies [71], [72] that have demonstrated that the choice of threshold can have a large influence on the topological properties of the binary graph. Moreover, our choice to employ a variable set of thresholds to obtain a fixed set of network densities ensures that our results are not be biased by individual or group differences in mean strength [73].

We quantified the organization of the binary networks using the path-length and clustering coefficient [74], global and local efficiency [14], [75], [76], betweenness centrality [77], modularity [78], [79], hierarchy [13], [80], synchronizability [81], [82], assortativity [13], [83], and robustness [15], [84]. In addition to topological network properties, we also studied the physical measure of mean connection distance [13], as well as a combined topophysical property Rent's exponent [85] which estimates the efficiency of the topological embedding of the network into physical space. Mathematical descriptions of these diagnostics are given in the SI.

### Statistical analyses

We tested for group differences in channel power, strength and entropy using a pairwise non-parametric permutation test (n = 10000 permutations) and we used the Holm-Bonferroni method for multiple comparisons correction. We examined the reliability of network properties over subjects and trials using the coefficient of variation (CV), which is defined as the standard deviation 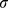 of a given sample normalized by its mean value 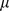:
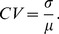
(1)


To test for inter-subject temporal variability in functional brain network organization, we calculated the CV for each network diagnostic, for each subject in both groups, and for all 10 intra- and inter-frequency networks. Note that we excluded the hierarchy and assortativity from this analysis because their values are close to zero, making the CV less meaningful and more prone to estimation errors from the division of numbers 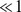. To test for group differences in temporal variability of network structure, we used a repeated measures ANOVA with CV values averaged over costs, group as a categorical factor, and frequency band and graph diagnostic as repeated measures.

To identify group differences in network diagnostics, we used Functional Data Analysis (FDA). FDA enables statistical inference from sets of functions [86] by extending the principles of statistical inference from data points to data curves. Here, in keeping with [20], the values of the graph properties were treated as a function of network density, and the two groups, (controls and people with schizophrenia) were compared with a non-parametric permutation test using twenty thousand permutations of group labels. With a total of 120 p-values (representing 10 networks – 4 intra- and 6 inter-frequency networks – times 12 graph properties), we chose to control for Type II errors due to multiple comparisons using a false discovery rate correction (FDR, 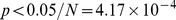).

In addition to testing for group differences in network structure, it is often of interest to determine whether the structure of an empirical network is different from what one would expect in a given null model. While the development of potentially useful network null models is ongoing [19], [87], here we employ benchmark Erdös-Renyi (ER) random graphs to test whether the network topology identified in intra- and inter-frequency functional brain networks was non-random. We created an ensemble of 66 ER graphs for each network density and each of the 4 intra- and 6 inter-frequency networks. Note that the number of networks in the ensemble was set to be identical to the number of networks in a single subject.

All computational and statistical operations were implemented in MATLAB® (2007a, The MathWorks Inc., Natick, MA). Network diagnostics were estimated using a combination of in-house software, the Brain Connectivity Toolbox [17], and the MATLAB Boost Graph Library [48]. The repeated measures ANOVA was performed using freely available code [88].

## Results

### Working memory performance

We examined the accuracy of performance in the 2-back working memory task for both the schizophrenia and control groups. People with schizophrenia had a significantly lower accuracy (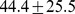
% (STD), the median was 56.1%) than controls (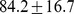
%): two sample t-test 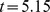, 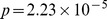. These results confirm that working memory function is impaired in our cohort of people with schizophrenia, supporting an additional investigation into the patterns of brain function during task performance.

### Time series variability and co-variability

#### Time series variability and co-variability

**Figure 1:**
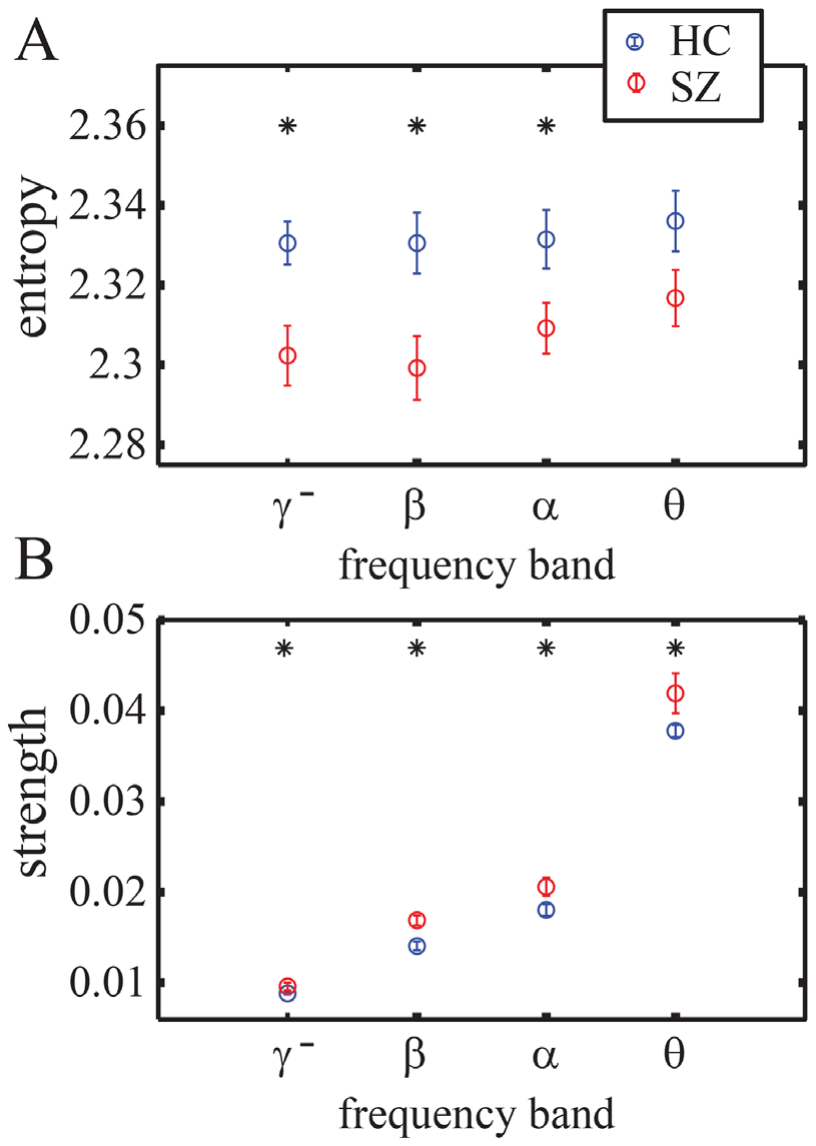
Group Differences in Activity and Connectivity. Wavelet entropy (*A*) and intra-frequency strength (*B*), averaged over sensors, in the 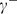-, 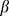-, 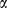
- and 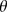-bands for healthy controls and people with schizophrenia. Asterisks indicate significant group differences as measured by non-parametric permutation tests (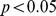, corrected for multiple comparisons using the Holm-Bonferroni method).

We examined the complexity of the MEG signals in the two groups by measuring the Shannon wavelet entropy of the sensor time series in four classical frequency bands (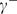-, 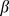-, 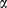
- and 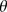; see Methods). On average, the entropy was lower in people with schizophrenia in 3 of the 4 bands (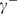, 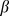, and 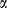); see Figure 1A.

The co-variability of sensor time series is thought to be a measurement of synchronous oscillatory neuronal activity and therefore potentially a proxy for large-scale communication between brain regions. We estimated the mutual information between sensor time series in the same four frequency bands (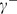-, 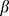-, 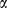
- and 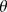; see Methods). The *strength* of functional connectivity of a sensor was defined as the average mutual information between that sensor's time series and the time series of all other sensors. Functional connectivity displayed frequency-dependent variation, being smallest in the highest frequency band (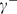) and largest in the lowest frequency band (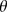) for both groups; see Figure 1. On average, functional connectivity was higher in people with schizophrenia all 4 bands (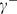, 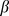, 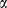, and 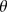). To rule out effects of differences in signal-to-noise ratio, we also estimated raw channel power for all individuals in this study and found no significant group difference (non-parametric permutation testing, 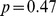).

#### Relationship between entropy and strength

**Figure 2:**
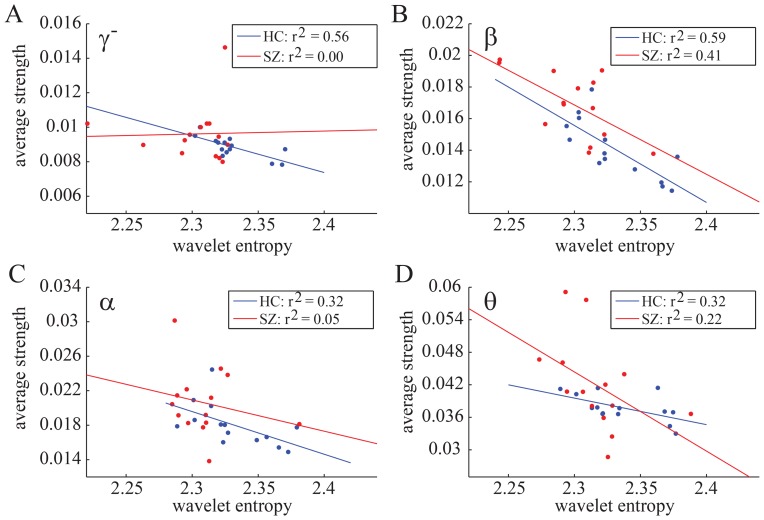
Entropy and Strength: Inter-Subject Level. Scatterplots of strength and complexity of the time series, as measured by wavelet entropy, for 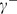-, 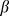-, 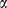
- and 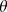-bands. Single data points represent values for each individual averaged over trials and sensors. Red markers denote subjects with schizophrenia spectrum diagnosis; blue markers denote healthy subjects. Lines indicate best linear fits for the two groups separately (red, SZ; and blue, HC) and we provide 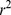 values as indicators of goodness of fit. Similar scatterplots that code each experimental block separately are provided in Figure S1 in the SI.

**Figure 3:**
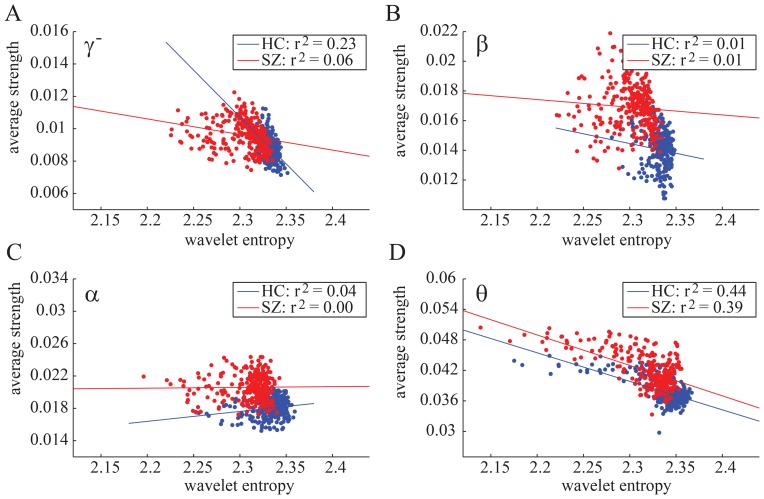
Entropy and strength: Sensor Level. Scatterplots of strength and wavelet entropy in 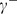-, 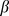-, 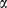
- and 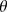-bands. Single data points represent values for each sensor averaged over trials and individuals. Red markers denote data from the SZ group; blue markers denote data from the HC group. Lines indicate linear fits for the two groups separately (red, SZ; and blue, HC) and we provide 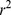 values as indicators of goodness of fit.

Recent work has described robust correlations between functional connectivity and estimates of time series complexity in fMRI activity measurements [19], [20], [38], which are present but altered in schizophrenia [20], [38]. Here we examine this potential relationship in a different imaging modality (MEG) both across frequency bands and between groups. In Figure 2, we show the strength and entropy of each individual, averaged over sensors and trials. Importantly, we find that correlations between the two measurements at the inter-subject level are frequency-dependent. Both groups display a significant inverse relationship between entropy and strength in the 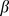-band, suggesting that individuals with high temporal variability in brain function have lower temporal co-variability. This relationship also appears to hold for the controls in the other frequency bands. In Figure 3, we show strength and entropy for each sensor (rather than each individual), averaged over trials and over individuals within a group. Again, we identify a strong inverse relationship between entropy and strength, which in this case is strongest in the 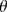-band. Of note, the inhomogeneous distribution of values characterized by a sparse low entropy tail is consistent with data reported in [20], and potentially indicates variation in the roles of brain areas in information processing.

### Network structure

Given the group differences in connectivity identified in the previous section, we next asked whether the patterns of connectivity between sensors are altered in schizophrenia. We characterize these patterns using binary network diagnostics. See Methods for details on binary network construction and the SI for mathematical definitions of network diagnostics.

#### Cost-efficiency

**Figure 4:**
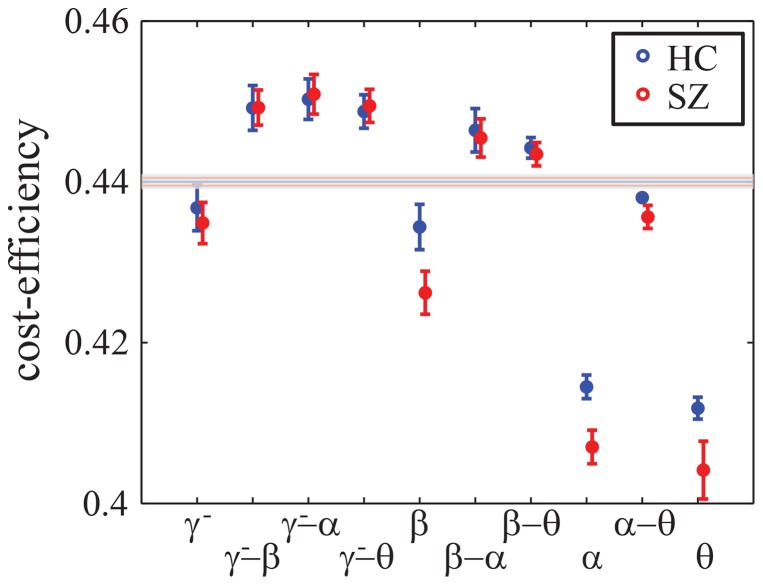
Cost-Efficiency of Functional Networks in Health and Disease. (*A*) Cost-efficiency in the 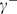-, 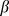-, 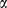-, 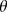-, and cross-frequency band networks for healthy controls (blue) and people with schizophrenia (red). Error bars indicate the standard error over subjects. The gray shaded line indicates the expected values of cost-efficiency for an ensemble of random (ER) graphs (see Methods).

We constructed binary graph diagnostics as a function of network density using a cumulative thresholding technique (see Methods). A simple network diagnostic that collapses such a curve into a single value is the cost-efficiency, which is defined as the maximum of the efficiency-minus-cost curve [14], [76], [89] (see the SI). In Figure 4A, we show the cost-efficiency of both intra- and inter-frequency networks for both groups. As expected from previous work [14], we found that cost-efficiency values decreased with increasing frequency. Importantly, intra-frequency networks demonstrated consistently lower cost-efficiency than inter-frequency networks. Group differences were only evident in intra-frequency networks (e.g., the 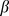, 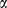 and 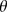 networks), with cost-efficiency being higher in the control group than in the patient group. Inter-frequency networks, while not demonstrating a group difference, did show a significantly higher cost-efficiency than expected in an ensemble of Erdös-Reny random graphs (see Methods), suggesting the presence of non-random structure in cross-frequency interactions.

#### Binary network organization

**Figure 5:**
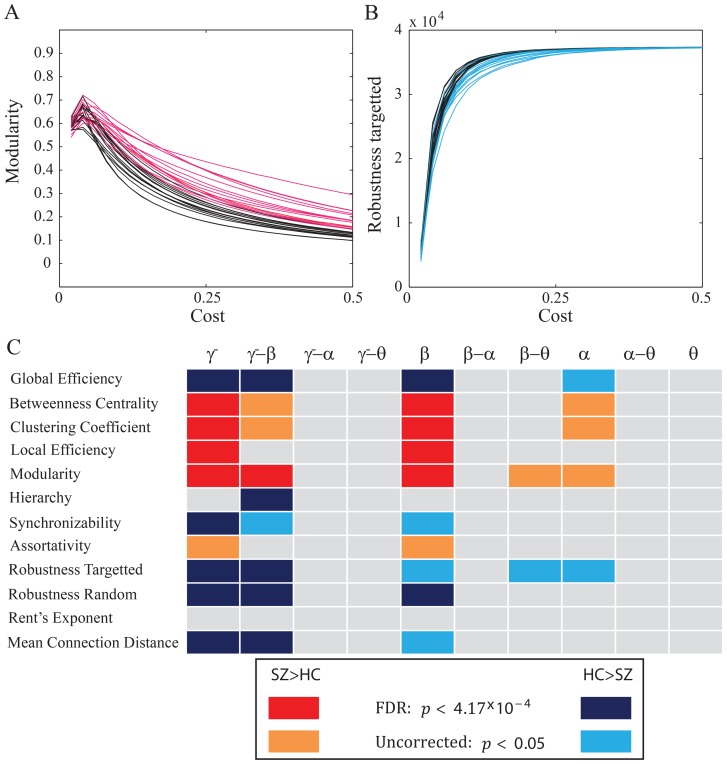
Binary Network Organization in Health and Disease. (*A*–*B*) Example binary network diagnostic curves as a function of threshold: the modularity for controls (HC; black) and people with schizophrenia (SZ; red) (*A*), and the robustness to targeted attack for controls (black) and people with schizophrenia (blue) for 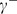-band networks. Individual curves indicate average values for each individual over the 66 trial-specific networks. (*C*) Significant group differences in graph diagnostic versus cost curves for 12 graph diagnostics (y-axis) in both intra- and inter-frequency bands (x-axis). Warm colors indicate that the diagnostics values were higher in people with schizophrenia than in healthy controls; cool colors indicate that the values were higher in healthy controls than in people with schizophrenia. Both warm and cool colors are two different shades corresponding to different levels of stringency for significance testing: false discovery rate (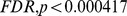), and uncorrected (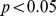). See Figures S2 and S3 in the SI for the full graph diagnostic versus cost curves for all subjects, frequency bands, and diagnostics.

While cost-efficiency has the advantage of collapsing a binary-diagnostic versus cost curve into a single value, it is also of interest to examine the shape of these curves for the other diagnostics. We examined 12 binary graph diagnostics as a function of cost for both groups and all 10 frequency bands [66]; see the Methods and the SI. We separated diagnostics into those that showed higher values in people with schizophrenia (e.g., see Figure 5A) and those that showed higher values in healthy controls (e.g., see Figure 5B). We found that the majority of significant group differences (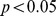 uncorrected) were located in the 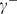 and 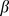 intra-frequency networks and in the 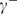-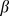 cross-frequency networks; see Figure 5C. The majority of these differences also pass corrections for multiple comparisons with the false discovery rate (FDR) method. In the 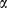-band, we also observed a trend for significant group differences in 5 out of the 12 diagnostics (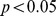 uncorrected).

People with schizophrenia displayed higher global and local efficiency, betweenness centrality, clustering coefficient, modularity, and assortativity and displayed lower hierarchy, synchronizability, mean connection distance and robustness to both targeted and random attack. In intra-frequency networks, these alterations are consistent with an abnormality in the distributed nature of healthy putative communication patterns present in adulthood [90], leading to a more local, more segregated, and less integrated information processing structure. In the 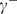-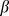 inter-frequency network, this pattern of results suggests that the integration of information over spectrally distinct processing streams [40], likely occurring between more distant brain areas, is impaired in schizophrenia, potentially impacting on working memory performance. More generally, the identification of group differences in both topological and physical network properties suggests that disease-associated changes in brain network organization might be linked to multiple developmental mechanisms. Indeed, recent theoretical work has suggested that altered constraints on both information efficiency (a topological property) and metabolic cost (a physical property) can lead to schizophrenia-like changes in network architectures [91].

#### Variability of binary network structure

Thus far we have reported results for each individual derived from networks constructed from 66 trial blocks. Here we ask whether network organization is variable over trial blocks in the two groups. To quantify temporal variability in network structure, we computed the coefficient of variation (CV) for network diagnostics over trial blocks for each individual in all 10 frequency bands.

**Figure 6:**
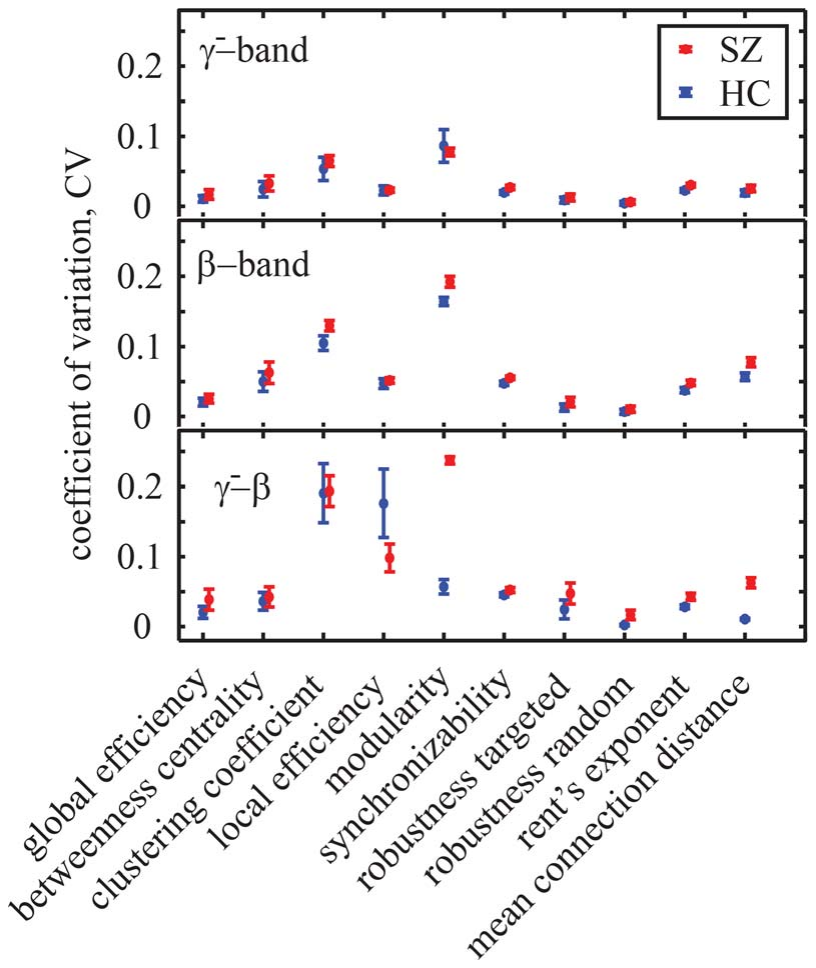
Temporal Variability of Network Diagnostics for binary network diagnostics in the 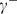 and 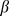 intra-frequency networks and in the 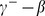 inter-frequency networks. CV values indicate temporal variability over trials for healthy (blue) and schizophrenic subjects (red), averaged over the entire range of cost values. Error bars indicate the mean squared error over subjects and costs. Values and error bars for synchronizability are scaled down by a factor of 10 for visualization purposes. Results for other frequency bands can be found in Figure S4 in the SI.

In Figure 6, we show the CV for the 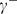 and 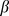 intra-frequency and the 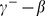 inter-frequency networks, averaged over costs (for similar results in other frequency bands, see the SI). We observe that the CV varies over binary graph diagnostics in a similar way across frequency bands. We also note that in almost all cases, the temporal variability appears to be larger for people with schizophrenia. To quantitatively test this observation, we used a Repeated Measures ANOVA (see Methods for details and Table 1 for results). The main effect of group (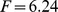, 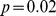) confirmed that the networks derived from people with schizophrenia varied more over time than did those derived from healthy controls, suggesting a fundamental alteration in the dynamics of functional brain networks in schizophrenia.

**Table 1:** Results for a Repeated Measures ANOVA of Temporal Variability in Network Topology, measured using the CV.

	SSE	DF	MSE	F	p
Group	0.04	1	0.04	6.24	0.02
Frequency Band	0.41	9	0.05	52.6	0
Diagnostic	6.24	9	0.69	309.74	0
Frequency Band*Diagnostic	1.63	81	0.02	41.83	0
Frequency Band*Group	0.04	9	0	4.52	1.68*e* ^−5^
Diagnostic*Group	0.11	9	0.01	5.27	1.52*e* ^−6^
Frequency Band* Diagnostic*Group	0.11	81	0	2.74	4.64*e* ^−14^

## Discussion

We have examined temporal characteristics of MEG data acquired from people with schizophrenia and controls during a working memory task. Our approach spanned several distinct levels, including that of the individual sensor time series (univariate), the co-variability between time series (bivariate), and the patterns of co-variability across sensors using binary network diagnostics (multivariate). We identified disease-associated changes in brain function at each level. People with schizophrenia displayed (1) lower time series entropy, suggestive of decreased information content of MEG signals and (2) higher strength of co-variability between time series, suggestive of hyperconnectivity between brain regions. Together, these results stand in contrast to the notion of schizophrenia as a disease characterized by reduced neurophysiological signal to noise ratios and reduced functional connectivity. We also found that people with schizophrenia displayed an extensive pattern of altered topological organization in binary sensor networks. Importantly, network properties of cross-frequency associations between time series in the 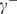
- and 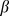-bands differed between groups, uncovering a novel feature of dysconnectivity. Finally, the temporal variability of brain network architecture in people with schizophrenia was significantly higher than that in healthy controls, a phenomenon suggestive of decreased dynamic constraints on brain function.

### 0.1. Implications for future studies

The mechanisms of psychotic symptoms in schizophrenia, their genetic underpinnings and their brain signatures, are far from understood. The interpretations of neuroimaging phenomena and related conclusions regarding neurophysiological mechanisms of the disease, are greatly hampered by the confounding effects of medication and disease heterogeneity associated with epiphenomena related to smoking and chronicity, as well as the heterogeneity of the mental state of ill subjects during these MEG procedures. Thus, it is impossible to conclude that our findings represent primary disease phenomena rather than epiphenomena related to secondary factors that are associated with the state of illness. These difficulties can be overcome to some degree by leveraging the genetic similarity between people with schizophrenia and their unaffected siblings and testing for similar imaging phenomena in both groups.

An important goal in schizophrenia research is the identification of intermediate phenotypes – observable characteristics that show tiered values in patients, their siblings, and controls – which might represent a powerful tool for the quantification of genetic risk mechanisms for psychiatric disease [92]. Alterations in brain functional architecture and temporal variability of brain activity have been proposed as such phenotypes [42], [93], [94] and might directly underlie the behavioral symptoms of psychosis [95]. Indeed, recent evidence suggests that functional connectivity might be more sensitive to genetics [96], [97] and to schizophrenia [20], [98] than the simple activity of brain regions alone.

Our extensive study of multiscale brain function in a small cohort of patients lays important groundwork for future studies that seek to uncover novel intermediate phenotypes by employing patients' siblings. Our results could serve to narrow the focus of such analyses to (1) high frequency 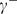 and 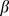 band functional connectivity, (2) network properties of whole-brain inter-frequency connectivity and (2) dynamic changes in network configuration during task performance.

### Altered time series variability

Signal variability or noise is a characteristic feature of the cortical system [99] and is thought to facilitate the exploration of functional network configurations necessary for healthy cognitive function [100]. Increased variability of stimulus-induced prefrontal electromagnetic activity has been observed in both people with schizophrenia and their healthy siblings [93], [101], [102], suggesting that cortical noise might be a genetic biomarker for the disease. Indeed, distributed patterns of both increased and decreased signal variability differentiate people with predominantly positive or negative symptom profiles [103], [104]. Furthermore, signal variability is behaviorally relevant, having been linked to task accuracy in both probands and healthy controls [93], [102], [105]. Increased high-frequency signal variability in people with schizophrenia has been observed in steady state evoked potentials [106] and resting state EEG [107]. Notably, in the latter study, this effect was most pronounced in frontal areas in drug-naive subjects and reduced by effective neuroleptic treatment.

Our results support the notion that signal variability is affected in schizophrenia. By employing the simple measure of time series entropy, we showed that signal variability was decreased in probands in high frequency bands (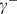 and 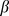). However, it is important to note that the Shannon entropy characterizes properties of the distribution of values in the time series rather than the temporal evolution of the signal. Alternative measures of temporal signal variability, such as the multi-scale entropy [108], the Renyi number [109], the Lyapunov exponent [110], the fractal dimension [103], [111], and the Hurst exponent [112], might provide greater or lesser sensitivity to disease state [113] or to disease-associated alterations in lower frequency bands.

### Activity and connectivity

Intuitively, one might imagine that the complexity of a region's time series would have some bearing on the strength of connectivity between that region and other regions. For example, a region with a more noisy signal might be less likely to show strong associations with other regions than a region with an ordered, strong oscillatory signal. Such a relationship would suggest that altered functional connectivity patterns can not only be driven by altered putative communication but also – more simply – by altered regional activity. Parsing the relative roles of region activity abnormalities and pairwise communication abnormalities is imperative for uncovering biological mechanisms of disease-related network changes.

A few recent neuroimaging studies address the relationship between activity and connectivity empirically. Three fMRI studies [19], [20], [38] and one MEG study [114] have reported a strong positive correlation between time series variability and co-variability during the resting state. However, our results suggest a more complex relationship in task-based data that is modulated by both frequency and disease. At the subject level, an inverse correlation between entropy and strength was evident in the 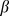 band for both groups, and also in the 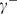, 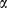 and 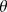 bands for the control group. Similarly at the sensor level, the entropy and strength were again inversely correlated, but in this case the effect was largest in the 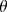 band. Importantly, the sensor-level analysis uncovered a strong heterogeneity in the distribution of entropy across sensors, complicating the statistical estimate of the entropy- strength relationship. Further work across imaging modalities and task states will be necessary to gain a greater intuition for the role of activity-connectivity relationships in cognitive function.

### Network structure of connectivity

Both binary and weighted network analyses each have their specific advantages and disadvantages and, when applied to the same data, can yield diverging results [72], [115]. Weighted networks suffer from issues of normalization, which are particularly important in the context of comparing groups with different average strength (as is the case here in our study), and further require the careful identification of appropriate null models [17], [19], [87]. Binary networks on the other hand rely heavily on the choice of thresholds, and neglect information contained in the weights of connections. However, binary networks are mathematically simpler [17] and their disadvantages can be at least partially overcome by examining diagnostic properties over a range of network densities. In this paper, we focus on binary networks and examine their properties over a wide and dense cost range. To identify group differences, we employ functional data analysis [20] and find that people with schizophrenia display higher local efficiency, betweenness centrality, clustering coefficient, modularity, and assortativity and display lower global efficiency, hierarchy, synchronizability, mean connection distance and robustness to both targeted and random attack than healthy controls in high frequency band networks.

Network organization in schizophrenia has been examined using a variety of structural (sMRI, DTI [12], [13], [116]) and functional (fMRI, EEG, and MEG) neuroimaging techniques [15], [18], [20], [29], [30], [117]–[120]. Schizophrenia-related alterations in network structure are reported across all imaging modalities and experimental paradigms. One seemingly modality-independent alteration [121] in schizophrenia is a reduction of global network efficiency (see [12], [116] for networks derived from diffusion imaging data, [18], [20], [118] for networks derived from resting state fMRI data, and [14] and our current study for networks derived from task-based MEG data). However, in general, the anatomical location, strength, and direction of these alterations can differ across brain states and between structural and functional imaging modalites (see Figure S5 in the SI for a summary visualization). This inherent complexity of the schizophrenia brain network signature is consistent with the hypothesis that transient state-related hypo-and hyper-connected functional profiles are superimposed on a general structural dysconnectivity [21]. Such a view is supported by recent work demonstrating that network organization changes with brain state, for example as a function of cognitive load [30], [122], learning [31], and multisensory stimulation [123]. Theoretical work using, for example, neural mass models [124]–[126] could potentially provide mechanistic descriptions of how state-dependent functional network alterations can stem from state-independent structural dysconnectivity. Such a theoretical mechanistic link would require empirical validation from multimodal imaging studies [127]–[130]. Moreover, it is worth emphasizing that evidence of gross structural dysconnectivity in schizophrenia, as suggested by imaging studies, is inconclusive as measures from structural MRI approaches are subject to diverse epiphenomena that confound interpretation.

### Dynamic network variability

We explored the evolution of functional connectivity during the performance of a working memory task. This exploration was guided by theoretical work proposing the critical role of functional network reconfigurations in healthy cognitive function [100] and by empirical work demonstrating the importance of reconfiguration flexibility for cognitive phenomena like learning [31]. We identified greater temporal network variability on average in people with schizophrenia than in healthy controls. This finding is consistent with theoretical formulations of dysconnectivity in schizophrenia that suggest a temporal disorganization of sequentially expressed dynamical states [131]. Indeed, from a theoretical point of view, the inability of the dynamic brain network to sustain the organization necessary for healthy cognitive function might empirically result in the fragmentation of cortical and subcortical networks seen in schizophrenia [132]. Empirically, our findings are also consistent with a recent EEG study by Schoen and colleagues [133] demonstrating an increased entropy of connections in schizophrenia specifically in the high frequency 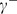 band. Together, these results point toward altered temporal trajectories of whole-brain cortical function potentially due to inadequate or altered constraints on brain dynamics in schizophrenia.

### Methodological limitations and considerations

In addition to intra-frequency investigations, we have constructed inter-frequency networks to determine whether cross-frequency communication patterns are altered in schizophrenia. Cross-frequency interactions are thought to facilitate the cross-function integration of information over spectrally distinct processing streams [40] and might be critical for memory function [39]. Phase-amplitude coupling and similar methods [41], [45], [134] have been used to quantify these interactions, but the theoretical foundations of these methods are still not well understood [135], [136]. We have chosen to use a relatively simple nonlinear measure of interaction – the mutual information between times series extracted from different wavelet bands – to quantify statistical associations between frequencies that could be studied from a network perspective. Although outside of the scope of the present paper, it would be interesting in future to examine the effects of alternative estimates of these interactions on network structure.

The data used for this study was acquired using a CTF machine, whose axial gradiometers have source profiles that include information from a wide spatial range, limiting the potential for anatomical interpretations. Commonly used source localization techniques allow for greater confidence in anatomical localization but simultaneously change the correlation structure between time series. Instead, to retain the inherent network correlation structure and increase the localization specificity, we transformed the data into planar space [14], [16]. With advanced setup and analysis techniques [50], source localization is however becoming a more reliable option and should be considered for future studies [137]. This however requires that head movements be both minimized during recording, which poses difficulties with some schizophrenic subjects especially, and corrected for during preprocessing. We did not perform such a correction, however, the effects of head movements on planar space trial-by-trial analysis of short trials, should be small.

In the present study, we did not observe significant correlations between the single-valued diagnostics (entropy, connectivity, cost-efficiency and variability) and the accuracy of task performance, for which our small sample size might be a factor. A more extensive examination of the relationship between behavioral variables and network diagnostics is outside of the scope of this study, whose focus was primarily to identify alterations in network structure and dynamics in schizophrenia.

Furthermore, as all of the people with schizophrenia included in this study were on medication, most of them were smokers, and their performance on the task was significantly worse than the performance of the normal subjects, we can not determine whether our findings are driven by the disease, by associated epiphenomena, as an effect rather than a cause of the poor task performance (e.g. altered attention, effort, distraction) or by medication or a combination of these. To examine the effects of these various epiphenomena, it would be important to perform a follow-up study in unaffected siblings, for which our study gives important guiding information.

## Conclusion

Recent advances in physics and mathematics have provided unique, robust quantitative network methods to examine the structure and organization of whole-brain functional connectivity. Mounting evidence from a plethora of imaging modalities, cognitive states, and diseases underscores the utility of network theory in capturing previously uninvestigated variations in large-scale brain function and its alteration in disease states such as schizophrenia [21], [43], [121]. In this study, we have introduced several new methods for studying dynamic properties of brain networks in schizophrenia, including cross-frequency networks and temporal variation in network structure. We report a multiresolution profile of alterations in activity, connectivity, network topology, and network dynamics that impacts on band-specific and cross-frequency putative communication patterns. Our study lays important methodological groundwork for future efforts in the identification of intermediate phenotypes [42] and in the development of diagnostic biomarkers [21], [43].

## Supporting Information

Figure S1
**Entropy and Strength.** Correlation between the strength of connectivity and complexity, as measured by wavelet entropy, in 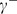-, 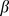-, 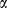
- and 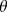-bands. Single data points represent the mean value pairs from a block of trials, trials from different subjects are distinguished by different colors and markers. Red, orange and pink markers denote subjects with schizophrenia spectrum diagnosis; blue, turquoise and purple markers denote healthy subjects. Value pairs from single subjects exhibit a tendency to appear in clusters, which (mostly) are broken up only at low entropy and/or high connectivity. With some subjects, value pairs appear outside the main cluster in all bands, with others, only in some. Also displayed are fitted linear functions for the two groups (red and blue lines) with 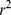 values as indicators of goodness of fit. It should be noted that these were obtained by fitting to the mean value pairs for subjects, averaged over all 6 blocks of trials. This was done to avoid fitting to values for which there are two sources of variance (subjects and blocks). These fits indicate a negative correlation between entropy and strength, especially for healthy subjects where 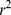 values are much higher.(EPS)

Figure S2
**Network Diagnostics: Part I.** One set of six network diagnostics (global efficiency, betweenness centrality, clustering coefficient, local efficiency, modularity, and hierarchy) is plotted as a function of density in networks within and between frequency bands. Each curve represents one subject, values averaged over all 66 trials. Curves for healthy controls are black, those for SZ patients colored. The two sets of curves were tested for statistically significant difference with Functional Data Analysis (FDA), the resulting p-values are given. Where significance (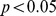) was calculated, the color of the SZ curves was set to red, purple otherwise. We see significant differences for most diagnostics between the groups in the 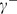, 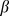 and 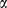 bands, as well as for the 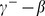 cross-frequency network.(EPS)

Figure S3
**Network Diagnostics: Part II.** A second set of six network diagnostics (synchronizability, assortativity, robustness to targeted and random attack, Rent's exponent, and mean connection distance) is plotted as a function of density in networks within and between frequency bands. Each curve represents one subject, values averaged over all 66 trials. Curves for healthy controls are black, those for SZ patients colored. The two sets of curves were tested for statistically significant difference with Functional Data Analysis (FDA), the resulting p-values are given. Where significance (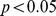) was calculated, the color of the SZ curves was set to red, purple otherwise. In the 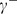 and 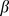 bands, as well as in the 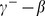 cross-frequency network, we see again a majority of diagnostics showing significant (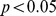) differences, but not in the 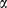 band.(EPS)

Figure S4 **Variability of network diagnostics.** Coefficient of variation for binary network diagnostics in all intra- and inter-frequency networks. Values indicate variability over trials, averaged over all healthy (blue) and schizophrenic subjects (blue) and over the entire range of cost values. Error bars indicate the square mean of the standard errors over subjects and costs.
(EPS)

Figure S5 **Comparison of Whole-Brain Network Metrics.** Results for a variety of network metrics are compared across different studies. (*Top Panel*): Resting-state fMRI studies [15], [18], [20], [118], [120], [138]. (*Middle Panel*) Structural studies [10], [12], [13], [116]. In Ref. [116], the metric are given for a fronto-parietal network only. (*Bottom Panel*): EEG and MEG studies [117], [139]–[142] and our present study (*Siebenh2013*). Results are given for each frequency band separately, where available. Different frequency bands are signified by different colors and different studies by different markers. Network diagnostics include functional connectivity (*Funct. Conn*), structural connectivity (*Connectivity*), clustering coefficient (*clustering*), modularity index (*modularity*), small-worldness (*SW-ness*), average path length (*path length*), local efficiency (*local eff*), global efficiency (*global eff*), cost efficiency (*cost eff*), mean connection distance (*MCD*), betweenness centrality (*centrality*), hierarchy parameter (*hierarchy*), robustness to random attack (*Robust rand*), and robustness to targeted attack (*Robust targ*). A data point outside (inside) the black circle indicates that the value was significantly higher (lower) in schizophrenic subjects than in healthy controls; a point on the circle indicates that the study explicitly stated that no significant difference was found. This figure only provides qualitative information; data points are given slightly different radial coordinates only for visibility, not to represent quantitative values.
(EPS)

Text S1 **Supplementary Information for “Intra- and Inter-Frequency Brain Network Structure in Health and Schizophrenia**.” This document includes mathematical definitions for network diagnostics employed in the present analysis, Figure S1–S5, and Tables S1–S2.
(PDF)

Table S1 **Duration of Illness and PANSS Scores.**
(PDF)

Table S2 **Medication Profile of Patient Group.**
(PDF)
