# Supplementary material for: Intra- and Inter-Frequency Brain Network Structure in Health and Schizophrenia
Source: PLoS One. 2013 Aug 26;8(8):e72351. doi: 10.1371/journal.pone.0072351 (PMC3753323; doi:10.1371/journal.pone.0072351)
Supplement: Text S1 — Supplementary Information for “Intra- and Inter-Frequency Brain Network Structure in Health and Schizophrenia.” This document includes mathematical definitions for network diagnostics employed in the present analysis, Figure S1–S5, and Tables S1–S2. (PDF) [file pone.0072351.s006.pdf]

Text S1: Supplementary Information for Intra- and Inter-Frequency Brain Network Structure in Health and Schizophrenia

Felix Siebenhühner[1,2], Shennan A. Weiss[3], Richard Coppola[4] Daniel R. Weinberger[5,6] Danielle S. Bassett[1,7,*]

1 Department of Physics, University of California, Santa Barbara, CA 93106 USA

2 Neuroscience Center, University of Helsinki, Helsinki, 00014 Finland

3 Department of Neurology, Columbia University New York, NY, 10027 USA

4 MEG Core Facility, National Institute of Mental Health, Bethesda, MD 20892 USA

5 Genes, Cognition and Psychosis Program, Clinical Brain Disorders Branch, National Institute of Mental Health, Bethesda, MD 20892 USA

6 Lieber Institute for Brain Development, Johns Hopkins Medical Campus, Baltimore, MD 21230 USA

7 Sage Center for the Study of the Mind, University of California, Santa Barbara, CA 93106 USA

∗ E-mail: Corresponding dbassett@physics.ucsb.edu

This supplementary materials document includes the following:

- Mathematical definitions for network diagnostics employed in the present analysis.
- Figure S1: Entropy and Strength
- Figure S2: Network Diagnostics: Part I
- Figure S3: Network Diagnostics: Part II
- Figure S4: Variability of Network Diagnostics: Part II
- Figure S5: Comparison of Whole-Brain Network Metrics
- Table S1: Duration of Illness and PANSS scores
- Table S2: Medication Profile of Patient Group

Mathematical Definitions

Time Series Diagnostics

Wavelet Entropy: Entropy, as defined by Shannon [1] is a simple, time-independent measure of the degree of order/disorder of the signal and has been applied to measure the univariate complexity of signals obtained in neuroimaging [2, 3]. Here, we calculated wavelet entropy with the MATLAB function *wentropy.m*:

$$E(s) = -\sum_i s_i^2 \log(s_i^2) \tag{1}$$

where s is the signal of a single region in a given individual and s_i are the coefficients of s in the orthonormal wavelet basis.

Network Diagnostics

A network is composed of units (*nodes*) and connections between those units (*edges*). The degree k_i of node i is defined as the number of edges emanating from node i.

Clustering coefficient The clustering coefficient C is defined by supposing that a node i has k_i neighbors, so a maximum of $k_i(k_i - 1)/2$ edges can exist between these neighbors [4]. The local clustering coefficient C_i is the fraction of these possible edges that actually exist:

$$C_i = \frac{\sum_{mj} A_{mj} A_{im} A_{ij}}{k_i(k_i - 1)} \,. \tag{2}$$

The clustering coefficient C of an entire network is then defined as the mean of C_i over all nodes i.

Hierarchy A sense of hierarchical structure of the network can be characterized by the coefficient β, which is a parameter quantifying the putative power law relationship between the clustering coefficient C_i and the degree k_i of all nodes in the network [5]:

$$C_i \sim k_i^{-\beta}. \tag{3}$$

Pragmatically, we estimate β using the best linear fit of C vs. k in loglog space with a robust outlier correction.

Assortativity The degree assortativity of a network (which is often called simply 'assortativity') is defined as

$$a = \frac{E^{-1}\sum_i j_i k_i - \left[E^{-1}\sum_i \frac{1}{2}(j_i + k_i)\right]^2}{E^{-1}\sum_i \frac{1}{2}(j_i^2 + k_i^2) - \left[E^{-1}\sum_i \frac{1}{2}(j_i + k_i)\right]^2} \,, \tag{4}$$

where j_i and k_i are the degrees of the nodes at the two ends of the ith edge ($i \in \{1, \ldots, E\}$ [6]. The assortativity measures the preference of a node to connect to other nodes of similar degree (leading to an assortative network, $r > 0$) or to other nodes of very different degree (leading to a disassortative network, $r < 0$). Social networks are commonly found to be assortative while networks such as the internet, World-Wide Web, protein interaction networks, food webs, and the neural network of C. elegans are disassortative.

Mean Connection Distance The estimated connection distance of an edge, $d_{i,j}$, is defined as the Euclidean distance between the centroids of the connected regions i and j in standard stereotactic space. The mean connection distance, d, is defined as the average connection distance over all edges in a network [7]. Thus connection distance differs from the other, topological and dimensionless network diagnostics in that it represents a spatial or topographic property of the network and has units of distance (mm).

Rent's exponent Rent's exponent is a topophysical property of a network; that is, it describes how a *non-physical* topology is embedded into a *physical* space, which in the case of neuronal fiber tracts is

the physical space of the brain [8]. Rent's rule, which was first discovered in relation to computer chip design, defines a scaling relationship between the number of external signal connections (edges) e to a block of logic and the number of connected nodes n in the block [9]:

$$e \sim n^p\,, \tag{5}$$

where $p \in [0,1]$ is the Rent exponent. Following [8], the Rent's exponent is found by tiling the Euclidean space of the network with $N_{\rm box} = 5000$ overlapping randomly sized boxes (e.g., three-dimension cubes). In each box we determine the number of nodes (n) and the number of connections (e) that cross the box boundaries. The gradient of a straight line fitted to $\log(n)$ versus $\log(e)$ using iteratively weighted least squares regression is an estimate of the Rent exponent p. To minimize boundary effects, p is estimated using the subset of boxes which contains less than half the total number of nodes, $n < N/2$.

Global efficiency The global efficiency was defined by Latora and Marchiori [10] and first applied to neuroimaging data in [11]. The regional efficiency of a single node, i, is defined as

$$E(i) = \frac{1}{N-1}\sum_{j\in G}\frac{1}{L_{i,j}}, \tag{6}$$

where $i = 1, 2, 3, \ldots, N$ indicates the index region, $j \neq i$ denotes a region connected to i, and $L_{i,j}$ is the minimum path length between regions i and j. Regional efficiency is therefore inversely related to minimum path length and a region with high efficiency will have short minimum path length to all other regions in the graph. The global efficiency of a graph is defined as the mean of $E(i)$ over all possible regions, and is commonly denoted E_{glob}.

Local efficiency Latora and Marchiori also defined a local efficiency, which measures the efficiency of the subgraph surrounding node i:

$$E(i) = \frac{1}{N_{G_i}(N_{G_i}-1)}\sum_{j,k\in G_i}\frac{1}{L_{j,k}}, \tag{7}$$

where G_i is the subgraph of nodes and edges connected to node i and $L_{j,k}$ is the minimum path length between nodes j and k in the subgraph [10].

Betweenness centrality Geodesic node betweenness or more simply 'betweenness centrality' is defined for the ith node in a network $\mathcal{G}$ as

$$B_i = \sum_{j,m,i\in\mathcal{G}}\frac{\psi_{j,m}(i)}{\psi_{j,m}}\,, \tag{8}$$

where all three nodes (j, m, and i) must be different from each other, $\psi_{j,m}$ is the number of geodesic paths between nodes j and m, and $\psi_{j,m}(i)$ is the number of geodesic paths between j and m that pass through node i. The betweenness centrality of an entire network B is defined as the mean of B_i over all nodes i in the network.

Modularity Networks can be partitioned into communities or modules [12, 13] where nodes inside the same community are more densely connected to each other than they are to nodes in other communities. The modularity [14, 6] of a network partition is defined as:

$$Q = \frac{1}{2m}\sum_{ij}[A_{ij} - \frac{k_i k_j}{2m}]\delta_{c_i,c_j}\,, \tag{9}$$

where k_i is the degree of node i, m is the total number of edges in the network, A_{ij} is an element of the adjacency matrix, δ_{ij} is the Kronecker delta symbol, and c_i is the label of the community to which node i has been assigned [15]. Here we used the Louvain locally greedy algorithm [16] to optimize the modularity quality function over the space of possible network partitions. We report the maximum value of Q over this optimization procedure.

Synchronizability The synchronizability, S, of a network characterizes structural properties of a graph that hypothetically enable it to synchronize rapidly [17]. The synchronizability is defined as

$$S = \frac{\lambda_2}{\lambda_N} \tag{10}$$

where λ_2 is the second smallest eigenvalue of the Laplacian $\mathcal{L}$ of the adjacency matrix, and λ_N is the largest eigenvalue of $\mathcal{L}$ [18].

Robustness The robustness metric, ρ, indicates the network's resilience to either targeted, ρ_t, or random, ρ_r, attack. In a targeted attack, hubs are removed one by one in order of degree, k, while in a random attack, nodes are removed at random independent of their degree. Each time a node was removed from the network, we re-calculated the size of the largest connected component, s. Robustness is then usually visualized by a plot of the size of the largest connected component, s, versus the number of nodes removed, n [19, 20]. The robustness parameter, ρ, is defined as the area under this s versus n curve. More robust networks retain a larger connected component even when several nodes have been knocked out, as represented by a larger area under the curve or higher values of ρ.

Cost Efficiency We define the cost efficiency [11, 21, 22] at a node as the maximal difference between the regional efficiency and the network density or cost over the investigated range of cost values:

$$CE(i) = E(i) - K. \tag{11}$$

We also define the cost efficiency of the network CE_{net} as the mean of $CE(i)$ over all nodes in the network.

References

[1] Shannon CE (1948) A mathematical theory of communication. Bell System Technical Journal 27: 379423.

[2] Rosso OA, Blanco S, Yordanova J, Kolev V, Figliola A, et al. (2001) Wavelet entropy: a new tool for analysis of short duration brain electrical signals. Journal of Neuroscience Methods 105: 65-75.

[3] Bassett DS, Nelson BG, Mueller BA, Camchong J, Lim KO (2012) Altered resting state complexity in schizophrenia. NeuroImage 59: 2196-207.

[4] Watts DJ, Strogatz SH (1998) Collective dynamics of 'small-world' networks. Nature 393: 440-442.

[5] Ravasz E, Barabasi A (2003) Hierarchical organization in complex networks. Phys Rev E 67: 026112.

[6] Newman MEJ (2006) Modularity and community structure in networks. Proc Natl Acad Sci USA 103: 8577-8582.

[7] Bassett DS, Bullmore ET, Verchinski BA, Mattay VS, Weinberger DR, et al. (2008) Hierarchical organization of human cortical networks in health and schizophrenia. J Neurosci 28: 9239-9248.

[8] Bassett DS, Greenfield DL, Meyer-Lindenberg A, Weinberger DR, Moore S, et al. (2010) Efficient physical embedding of topologically complex information processing networks in brains and computer circuits. PLoS Comput Biol 6: e1000748.

[9] Christie P, Stroobandt D (2000) The interpretation and application of Rent's Rule. IEEE Trans VLSI Syst 8: 639-648.

[10] Latora V, Marchiori M (2001) Efficient behavior of small-world networks. Phys Rev Lett 87: 198701.

[11] Achard S, Bullmore E (2007) Efficiency and cost of economical brain functional networks. PLoS Comput Biol 3: e17.

[12] Porter MA, Onnela JP, Mucha PJ (2009) Communities in networks. Not Amer Math Soc 56: 1082–1097, 1164–1166.

[13] Fortunato S (2010) Community detection in graphs. Phys Rep 486: 75–174.

[14] Girvan M, Newman MEJ (2002) Community structure in social and biological networks. Proc Natl Acad Sci USA 99: 7821–7826.

[15] Leicht EA, Newman MEJ (2008) Community structure in directed networks. Phys Rev Lett 100: 118703.

[16] Blondel VD, Guillaume JL, Lambiotte R, Lefebvre E (2008) Fast unfolding of communities in large networks. Journal of Statistical Mechanics: Theory and Experiment 2008: P10008.

[17] Bassett DS, Meyer-Lindenberg A, Achard S, Duke T, Bullmore E (2006) Adaptive reconfiguration of fractal small-world human brain functional networks. Proc Natl Acad Sci USA 103: 19518-19523.

[18] Barahona M, Pecora L (2002) Synchronization in small world systems. Phys Rev Lett 89: 054101.

[19] Achard S, Salvador R, Whitcher B, Suckling J, Bullmore E (2006) A resilient, low-frequency, small-world human brain functional network with highly connected association cortical hubs. J Neurosci 26: 63-72.

[20] Lynall ME, Bassett DS, Kerwin R, McKenna P, Muller U, et al. (2010) Functional connectivity and brain networks in schizophrenia. J Neurosci 30: 9477-9487.

[21] Bassett DS, Meyer-Lindenberg A, Weinberger DR, Coppola R, Bullmore E (2009) Cognitive fitness of cost-efficient brain functional networks. Proc Natl Acad Sci USA 106: 11747-52.

[22] Fornito A, Zalesky A, Bassett D, Meunier D, Ycel M, et al. (2011) Ggenetic influences on cost-efficient organization of human cortical functional networks. J Neurosci 31: 3261-70.

[23] Yu Q, Plis SM, Erhardt EB, Allen EA, Sui J, et al. (2012) Modular organization of functional network connectivity in healthy controls and patients with schizophrenia during the resting state. Frontiers in Systems Neuroscience 5.

[24] Yu Q, Sui J, Rachakonda S, He H, Gruner W, et al. (2011) Altered topological properties of functional network connectivity in schizophrenia during resting state: A small-world brain network study. PLoS ONE 6: e25423.

[25] Liu Y, Liang M, Zhou Y, He Y, Hao Y, et al. (2008) Disrupted small-world networks in schizophrenia. Brain 131: 945-961.

[26] Alexander-Bloch AF, Vértes PE, Stidd R, Lalonde F, Clasen L, et al. (2013) The anatomical distance of functional connections predicts brain network topology in health and schizophrenia. Cereb Cortex 23: 127-138.

[27] van den Heuvel MP, Mandl RC, Stam CJ, Kahn RS, Hulshoff Pol HE (2010) Aberrant frontal and temporal complex network structure in schizophrenia: a graph theoretical analysis. J Neurosci 30: 15915-26.

[28] Zalesky A, Fornito A, Seal ML, Cocchi L, Westin CF, et al. (2011) Disrupted axonal fiber connectivity in schizophrenia. Biol Psychiatry 69: 80-9.

[29] Wang Q, Su TP, Zhou Y, Chou KH, Chen IY, et al. (2012) Anatomical insights into disrupted small-world networks in schizophrenia. NeuroImage 59: 1085 - 1093.

[30] Micheloyannis S, Pachou E, Stam CJ, Breakspear M, Bitsios P, et al. (2006) Small-world networks and disturbed functional connectivity in schizophrenia. Schizophrenia Research 87: 60-66.

[31] Rubinov M, Knock SA, Stam CJ, Micheloyannis S, Harris AW, et al. (2009) Small-world properties of nonlinear brain activity in schizophrenia. Hum Brain Mapp 30: 403-16.

[32] De Vico Fallani F, Maglione A, Babiloni F, Mattia D, Astolfi L, et al. (2010) Cortical network analysis in patients affected by schizophrenia. Brain Topography 23: 214-220.

[33] Uhlhaas PJ, Singer W (2006) Neural synchrony in brain disorders: Relevance for cognitive dysfunctions and pathophysiology. Neuron 52: 155-168.

[34] Spencer KM, Nestor PG, Niznikiewicz M, Salisbury D, Shenton M, et al. (2003) Abnormal neural synchrony in schizophrenia. J Neurosc 23: 7407-7411.

Figure and Table Legends

Figure S1. **Entropy and Strength.** Correlation between the strength of connectivity and complexity, as measured by wavelet entropy, in γ^--, β-, α- and θ-bands. Single data points represent the mean value pairs from a block of trials, trials from different subjects are distinguished by different colors and markers. Red, orange and pink markers denote subjects with schizophrenia spectrum diagnosis; blue, turquoise and purple markers denote healthy subjects. Value pairs from single subjects exhibit a tendency to appear in clusters, which (mostly) are broken up only at low entropy and/or high connectivity. With some subjects, value pairs appear outside the main cluster in all bands, with others, only in some. Also displayed are fitted linear functions for the two groups (red and blue lines) with r^2 values as indicators of goodness of fit. It should be noted that these were obtained by fitting to the mean value pairs for subjects, averaged over all 6 blocks of trials. This was done to avoid fitting to values for which there are two sources of variance (subjects and blocks). These fits indicate a negative correlation between entropy and strength, especially for healthy subjects where r^2 values are much higher.

Figure S2. **Network Diagnostics: Part I** One set of six network diagnostics (global efficiency, betweenness centrality, clustering coefficient, local efficiency, modularity, and hierarchy) is plotted as a function of density in networks within and between frequency bands. Each curve represents one subject, values averaged over all 66 trials. Curves for healthy controls are black, those for SZ patients colored. The two sets of curves were tested for statistically significant difference with Functional Data Analysis (FDA), the resulting p-values are given. Where significance ($p < 0.05$) was calculated, the color of the SZ curves was set to red, purple otherwise. We see significant differences for most diagnostics between the groups in the γ^-, β and α bands, as well as for the $\gamma^- - \beta$ cross-frequency network.

Figure S3. **Network Diagnostics: Part II** A second set of six network diagnostics (synchronizability, assortativity, robustness to targeted and random attack, Rent's exponent, and mean connection distance) is plotted as a function of density in networks within and between frequency bands. Each curve represents one subject, values averaged over all 66 trials. Curves for healthy controls are black, those for SZ patients colored. The two sets of curves were tested for statistically significant difference with Functional Data Analysis (FDA), the resulting p-values are given. Where significance ($p < 0.05$) was calculated, the color of the SZ curves was set to red, purple otherwise. In the γ^- and β bands, as well as in the $\gamma^- - \beta$ cross-frequency network, we see again a majority of diagnostics showing significant ($p < 0.05$) differences, but not in the α band.

Figure S4. **Variability of network diagnostics.** Coefficient of variation for binary network diagnostics in all intra- and inter-frequency networks. Values indicate variability over trials, averaged over all healthy (blue) and schizophrenic subjects (blue) and over the entire range of cost values. Error bars indicate the square mean of the standard errors over subjects and costs.

Figure S5. **Comparison of Whole-Brain Network Metrics.** Results for a variety of network metrics are compared across different studies. *(Top Panel)*: Resting-state fMRI studies [3, 23, 24, 25, 20, 26]. *(Middle Panel)* Structural studies [7, 27, 28, 29]. In Ref. [29], the metric are given for a fronto-parietal network only. *(Bottom Panel)*: EEG and MEG studies [30, 31, 32, 33, 34] and our present study (*Siebenh2013*). Results are given for each frequency band separately, where available. Different frequency bands are signified by different colors and different studies by different markers. Network diagnostics include functional connectivity (*Funct. Conn.*), structural connectivity (*Connectivity*), clustering coefficient (*clustering*), modularity index (*modularity*), small-worldness (*SW-ness*), average path length (*path length*), local efficiency (*local eff.*), global efficiency (*global eff.*), cost efficiency (*cost eff.*), mean con-

nection distance (*MCD*), betweenness centrality (*centrality*), hierarchy parameter (*hierarchy*), robustness to random attack (*Robust rand*), and robustness to targeted attack (*Robust targ*). A data point outside (inside) the black circle indicates that the value was significantly higher (lower) in schizophrenic subjects than in healthy controls; a point on the circle indicates that the study explicitly stated that no significant difference was found. This figure only provides qualitative information; data points are given slightly different radial coordinates only for visibility, not to represent quantitative values.

Table S1. **Duration of Illness and PANSS scores.**

Table S2. **Medication Profile of Patient Group.**
