# Supplementary material for: Intra- and Inter-Frequency Brain Network Structure in Health and Schizophrenia
Source: PLoS One. 2013 Aug 26;8(8):e72351. doi: 10.1371/journal.pone.0072351 (PMC3753323; doi:10.1371/journal.pone.0072351)
Supplement: Table S1 — Duration of Illness and PANSS Scores. (PDF) [file pone.0072351.s007.pdf]

Table 1: Duration of Illness and PANSS scores

	S1	S2	S3	S4	S5	S6	S7	S8	S9	S10	S11	S12	S13	S14
Duration (years)	5	5	9	26	6	7	6	5	10	20	14	31	13	21
Positive Symptoms														
Delusions	4	1	4	1	3	1	2	2	5		3	1	1	1
Conceptual	3	5	1	5	1	1	1	1	3	5	2	1	6	1
Hallucinatory	5	1	5	3	1	1	1	4	1	1	7	5	5	3
Excitement	2	1	1		1	1	1	1	2	1	2	1	1	1
Grandiosity	1	1	1	1	1	1	1	4	3	1	7	1	1	1
Suspiciousness	3	4	3	1	3	1	1	3	3	6		6	4	3
Hostility	1	1	1	1	1	1	1	1	3	4	1	6	4	1
**Sum**	**19**	**14**	**16**	**12**	**11**	**7**	**8**	**16**	**20**	**18**	**22**	**21**	**22**	**11**
Negative Symptoms														
Blunted affect	4	6	3	4	5	1	1	1	2		3	1	4	4
Emotional withdrawal	4	6	3	4	4	1	4	1	3		3	3	5	4
Poor rapport	4	5	2	4	4	1	1	1	6		2	1	4	3
Passiveness	4	6	3	4	4	1	4	1	2		2	3	3	3
Abstract thinking	4	1	2	5	5	1	1	1	3		6	1	6	1
Lack of Spontaneity	4	7	3	5	4	1	1	1	2		4	1	6	3
Stereotyped thinking	2	1	1	1	1	1	1	1	2		1	1	1	1
**Sum**	**26**	**32**	**17**	**27**	**27**	**7**	**13**	**7**	**20**	**0**	**21**	**11**	**29**	**19**
General Symptoms														
Somatic	2	1	2	1	1	1	1	6	1	4	2	5	3	4
Anxiety	1	1	3	1	3	2	4	1	3	5	7	6	1	6
Guilt	1	1	2	1	3	1	1	1	6	1	1	5	1	4
Tension	1	1	3	1	3	2	1	1	2	4	1	2	1	4
Mannerisms	1	4	1	1	1	1	1	1	1	1	1	1	1	1
Depression	5	3	3	1	1	1	1	1	5	3	1	6	1	2
Motor retardation	4	5	1	3	1	2	1	1	1	4	2	1	3	1
Uncooperativeness	1	1	1	1	1	1	1	1	6	3	1	1	1	1
Unusual thoughts	4	1	3	3	2	1	1	6	2	5	7	1	1	4
Disorientation	1	1	1	1	1	1	5	1	1	1	1	1	1	1
Poor attention	3	1	3	1	4	2	1	1	2	1	1	1	1	1
Lack of judgment	4	5	2	4	1	1	1	1	7	5	5	1	4	5
Disturbed volition	2	1	3	1	4	1	1	1	3	1	1	5	4	1
Poor impulse control	1	1	2	1	1	1	1	1	3	1	1	1	1	1
Preoccupation	4	4	2	1	1	1	1	2	2	6	7	1	1	5
Social avoidance	3	5	3	1	3	2	1	1	2	5	1	5	1	1
**Sum**	**38**	**36**	**35**	**23**	**31**	**21**	**23**	**27**	**47**	**50**	**40**	**43**	**26**	**42**
Additional Symptoms														
Anergia	13	18	8	12	11	5	11	4	7		9	6	13	10
Thought Disturbance	13	8	10	12	5	4	4	15	9	12	23	8	13	9
Activation	4	6	5		5	4	3	3	5	6	4	4	3	6
Paranoid/Belligerence	5	6	5	3	5	3	3	5	12	13		13	9	5
Depression	9	6	10	4	8	5	7	9	15	13	11	22	6	16
