# Supplementary material for: Intra- and Inter-Frequency Brain Network Structure in Health and Schizophrenia
Source: PLoS One. 2013 Aug 26;8(8):e72351. doi: 10.1371/journal.pone.0072351 (PMC3753323; doi:10.1371/journal.pone.0072351)
Supplement: Table S2 — Medication Profile of Patient Group. (PDF) [file pone.0072351.s008.pdf]

Table 1: Medication Profile of Patient Group. Values are given in units of milligrams per day.

	S1	S2	S3	S4	S5	S6	S7	S8	S9	S10	S11	S12	S13	S14
Zyprexa	15					20	20			30				
Aripiprazole		30	10					30			15			
Clozapine					300								350	600
Haldol	15													
Cogentin	5		2											
Lithium			600											
Ambien			5											
Geodon			160											
Prozac					20									
Paxil								25						
Seroquel								800						
Zoloft										100				
Ativan										1.5				
Ambien										10		10		
Risperdal											2.5			
Geodon											160			
Luvox											150			
Remeron												90		
Valium												30		
Klonopin														4
Thorazin														400
